# Breastfeeding Measurement - How Do We Define and Measure Breastfeeding Cessation Across Scientific Disciplines?

**DOI:** 10.1177/08903344251382505

**Published:** 2025-10-14

**Authors:** M. A. Theurich, J. Gencel-Augusto, M. S. Butler, L. Fischer, Z. T. Haile, E. Chetwynd

**Affiliations:** 1 Ludwig-Maximilians-Universität München (LMU Munich), Munich, Germany; 2 Pettenkofer School of Public Health, Munich, Germany; 3 Department of Preventive Medicine, Northwestern University Feinberg School of Medicine, Chicago, IL, USA; 4 Department of Social Medicine, Ohio University Heritage College of Osteopathic Medicine, Dublin, OH, USA; 5 School of Medicine, Department of Family Medicine, University of North Carolina at Chapel Hill, Chapel Hill, NC, USA

**Keywords:** breastfeeding, breastfeeding assessment, breastfeeding assessment instruments, breastfeeding cessation, breastfeeding discontinuation, breastfeeding termination, maternal psychology, weaning

## Abstract

In an earlier paper, we summarized the meanings of the term “weaning” as it is used colloquially, clinically, and in scientific and grey literature. Due to the various potential definitions, we suggested that researchers avoid using the term and instead aim to use standard terms and definitions that more precisely describe the specific transition in infant and young child feeding they are referring to. One key concept from the first paper is “breastfeeding cessation.” In this subsequent article, we go a step further to more precisely define breastfeeding cessation, guiding how to choose the level of its measurement. We discuss potential parameters, proxies for breastfeeding cessation, measurement tools, and instruments pertinent to the measurement and monitoring of breastfeeding cessation in qualitative and quantitative research. This paper draws on perspectives from different scientific disciplines, including clinical research, public health, psychology, and anthropology. By doing so, we aim to deepen appreciation for tools and instruments used across these disciplines, ultimately fostering a common understanding of parameters, tools, and approaches for measuring breastfeeding cessation.

## Introduction

In an earlier paper, we summarized the meanings of the term “breastfeeding cessation” to encompass either (1) the cessation of direct breastfeeding, defined as ceasing to put a child to the breast; or (2) the cessation of any human milk feeding, defined as ending the provision of any form of human milk (Theurich et al., 2025). In this paper, our objective is to further define breastfeeding cessation as a concept—including potential levels of measurement—and give examples of instruments and tools for monitoring and measuring it.

A recent scoping review on terminology used synonymously with “breastfeeding cessation” in empirical literature found several papers describing breastfeeding cessation as the “discontinuation of breastfeeding” (Batterjee & Zedan, 2024). While the discontinuation of breastfeeding can be empirically defined and measured, deciding on which measurement approaches to use in research can be challenging. The following sections outline how to precisely define breastfeeding cessation as it relates to the research question, explaining the potential levels of measurement and corresponding measurement tools.

## Define the Timing of Breastfeeding Cessation

After defining breastfeeding cessation as the “discontinuation of direct breastfeeding” or as “the cessation of any human milk feeding,” the second important facet to define is the timing, or age at which breastfeeding stops. Researchers may choose to categorize the timing of breastfeeding cessation as either: (1) premature or (2) timely. Some research studies refer to “premature” or “early” breastfeeding cessation, while others refer to “early, unplanned” cessation (Bookhart et al., 2025). For our purposes, we suggest defining premature breastfeeding cessation as the discontinuation of breastfeeding prior to public health recommendations, whether or not that cessation is planned or unplanned.

Timely breastfeeding cessation refers to termination of breastfeeding at an age in line with public health recommendations (e.g., without any known or expected health deficits to the mother or child). Specific recommendations for timely breastfeeding cessation according to global World Health Organization (WHO) recommendations (up to 2 years of age or beyond) were outlined in our previous paper (Theurich et al., 2025). It is important that researchers define which public health recommendations they are using within their studies as a benchmark for classifying breastfeeding cessation as either premature or timely.

## Define the Etiology of Breastfeeding Cessation

Timely breastfeeding cessation is considered a physiological process in healthy populations, which is not driven by pathologies, underlying medical conditions, or clinical breastfeeding difficulties in the infant or mother. Physiological breastfeeding cessation has not been studied in clinical research as often as premature breastfeeding cessation. Literature on the optimal age for breastfeeding cessation in early childhood is mostly viewed as a psychological decision between the mother and child, which is rooted in child development, bonding, and independence (Corsello & Agostoni, 2024). In anthropological research, scientists have measured the age at breastfeeding termination in prehistoric populations to approximate a time window for physiological breastfeeding cessation (Dąbrowski et al., 2020).

In clinical research, determining the etiology of premature breastfeeding cessation is a central research question. Premature breastfeeding cessation is often used as an outcome (dependent) variable in studies examining medical conditions or biopsychosocial factors that disrupt the breastfeeding relationship. However, breastfeeding cessation is not synonymous with mammary gland dysfunction. The term “lactation failure” may encompass various atypical or dysfunctional states of the mammary gland and is used synonymously with the terms lactation dysfunction, lactation insufficiency, insufficient milk syndrome, agalactia, hypogalactia, hypoplasia, or inadequate glandular tissue (Batterjee & Zedan, 2024). These states may precede premature breastfeeding cessation but are not synonymous with it.

Similarly, breastfeeding cessation is not synonymous with the lactational stage. While breastfeeding cessation precedes mammary involution, the term “breastfeeding cessation” describes the waning of a breastfeeding relationship, whereas “mammary involution” describes the physiological process of the mammary gland returning to its non-lactating stage. The developmental stages of the mammary gland include: embryonic, prepubertal, pubertal, pregnancy, lactation, and involution (Jena et al., 2019). Mammary involution is the physiological process by which the breast returns to its non-lactational stage, characterized by mammary epithelial cell apoptosis, matrix remodeling, and the generation of cells (Jena et al., 2019). A woman with functional lactation physiology who stops breastfeeding may simultaneously, and oftentimes, still have copious milk production and may therefore not yet be undergoing mammary involution.

**Key Messages**

- By precisely defining breastfeeding cessation and offering approaches for measurement, this article promotes consistency across scientific studies.
- Breastfeeding cessation can refer to the end of direct breastfeeding or the complete cessation of human milk feeding.
- The timing of breastfeeding cessation should be measured and classified as “premature” or “timely” with reference to established guidelines.
- Consider measuring the time span of breastfeeding cessation, starting from the initial attenuation in breastfeeding intensity and ending with breastfeeding termination.
- Choose to study physiological breastfeeding cessation or the etiology of premature breastfeeding cessation, like pathology or psychosocial variables.
- Choose a measurement level for breastfeeding cessation, deciding to measure it as a dichotomous, ordinal, or continuous variable.

In summary, breastfeeding cessation refers to an attenuation in the breastfeeding relationship, and not to mammary functional status or the lactational stage. Therefore, researchers should aim to be precise when using these various terms.

## Measure the Duration of Breastfeeding Cessation

**Figure 1.**
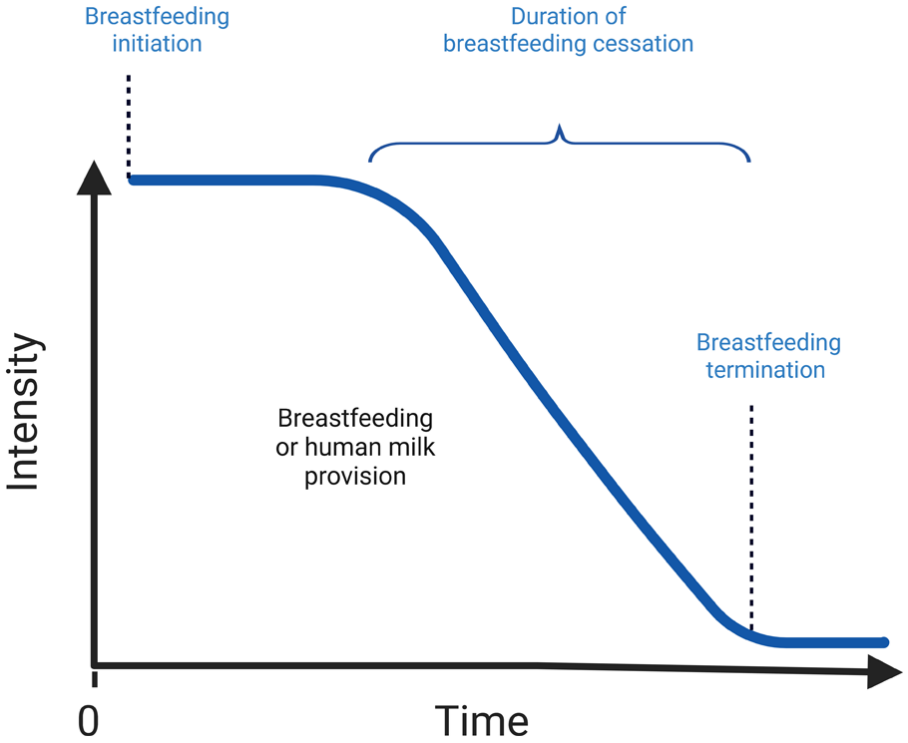
Breastfeeding Cessation as a Function of Breastfeeding Intensity Over Time.

While some breastfeeding relationships stop abruptly, others may ebb over time. “Breastfeeding duration” is the time span that begins with the breastfeeding initiation, usually at birth, and that ends when breastfeeding stops. However, the concept of the “duration of breastfeeding cessation” as a physiological process has not been extensively studied. Duration of breastfeeding cessation can be defined as the time span that begins with a decline in breastfeeding intensity and that ends when breastfeeding (or human milk feeding) fully stops. The concept of breastfeeding intensity should be precisely defined by researchers in their study (e.g., total human milk feedings, total milk volume, etc.; see Figure 1).

## Decide the Level of Measurement

**Figure 2.**
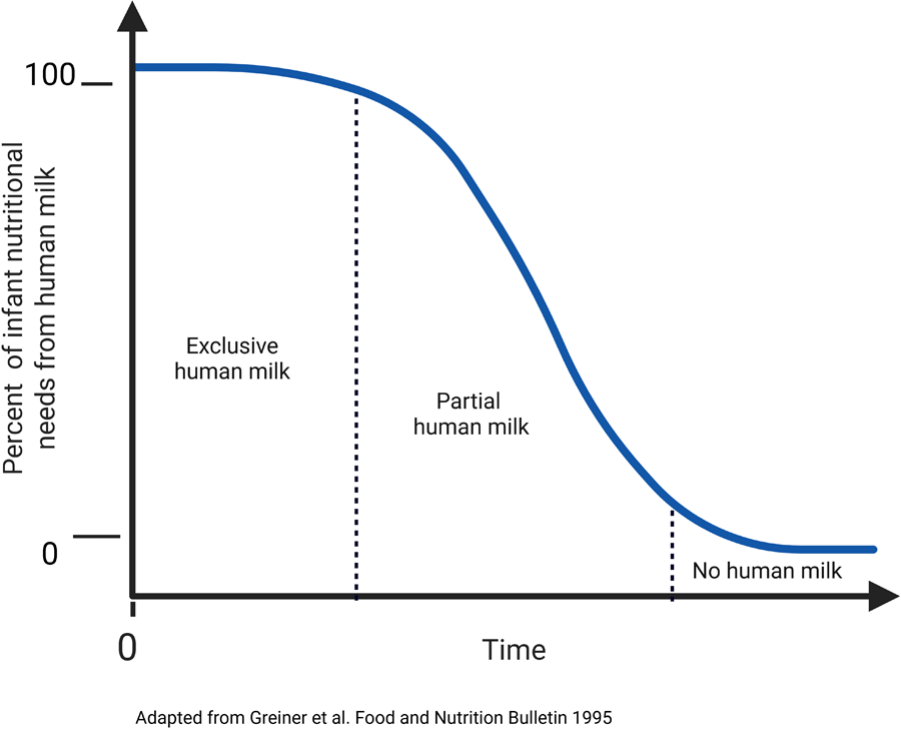
Breastfeeding Cessation as an Ordinal or Continuous Variable.

In clinical research, breastfeeding cessation can be measured as a dichotomous variable (e.g., “breastfeeding” or “discontinued breastfeeding”), an ordinal variable (e.g., “breastfeeding,” “partial cessation,” “full cessation”), or a continuous variable, which allows visualizing trends or patterns of the cessation process (e.g., gradual attenuation of total human milk volume or gradual attenuation of the percent of dietary energy from human milk over time). Choosing which level of measurement to use is driven by the research question and outcome of interest. Figure 2 provides a schematic illustration of how human milk can be measured as either an ordinal or a continuous variable.

**Figure 3.**
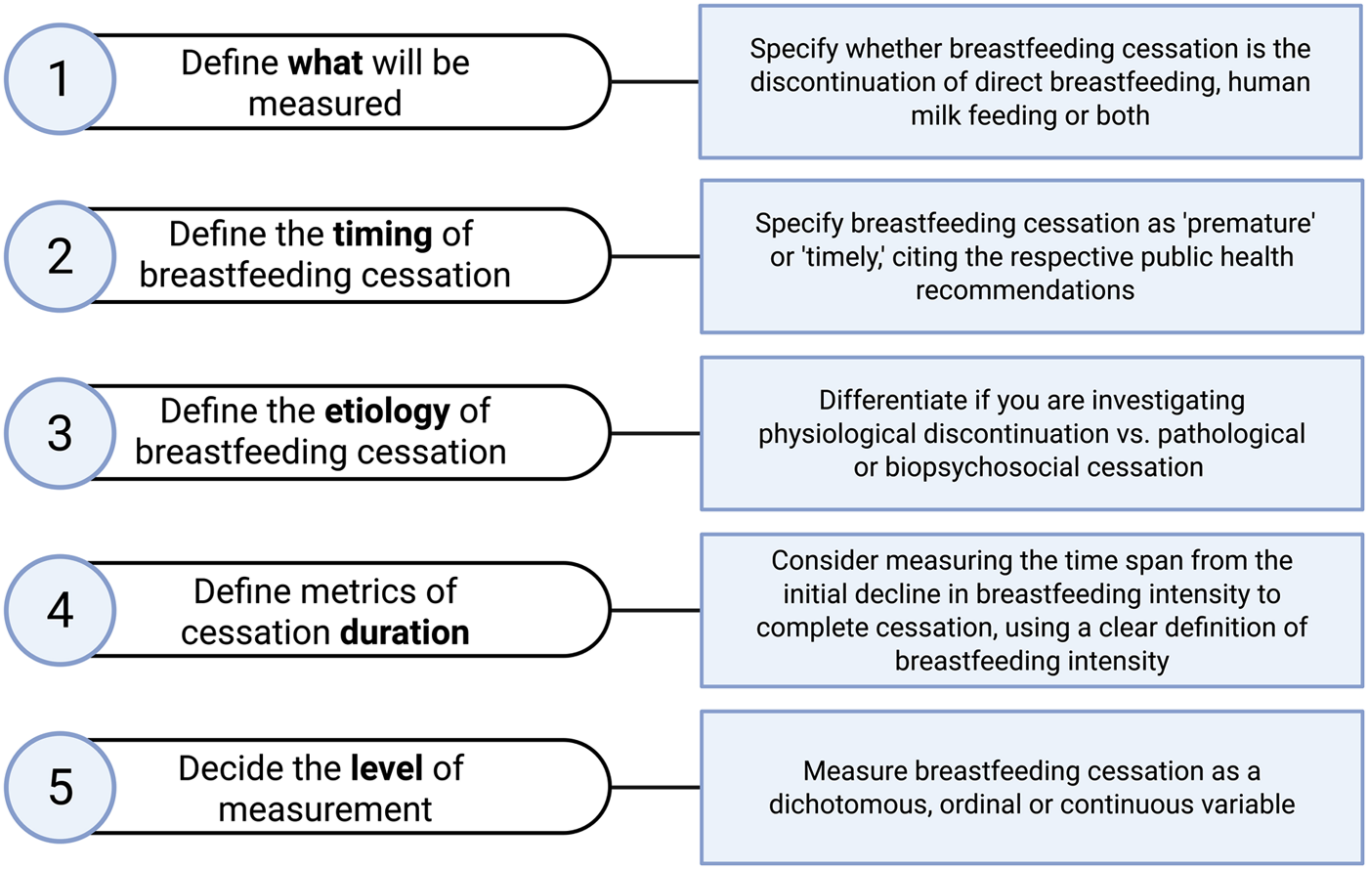
Considerations for Measuring Breastfeeding Cessation.

In summary, aim to use a precise definition of breastfeeding cessation, specifying whether direct breastfeeding or human milk feeding is being discontinued, describe the timing, etiology, and duration of breastfeeding cessation, and decide the level of measurement (see Figure 3).

## Measurement Tools and Instruments

In the following sections, available measurement tools are drawn from various scientific disciplines. The tools outlined in the following sections are not an exhaustive list and should not be considered endorsed by the authors. While some tools may be specific to one discipline, others may be applied across scientific disciplines or may be embedded between research and clinical care.

## Measurement Tools and Instruments From Clinical Research

Clinical research on breastfeeding and human lactation may come from medical or allied health specialties and subspecialties. To study breastfeeding cessation, clinical researchers may measure the attenuation in breastfeeding directly (milk volume, milk transfer) or indirectly (changes in feeding schedules, infant growth).

### Maternal Milk Volume

Measuring maternal milk volume has been previously used to track maternal milk volume over time, evaluate the function of the mammary gland, and determine transitions between lactation stages (Meier et al., 2016; Roznowski et al., 2020; Scanlon et al., 2002). Maternal milk volume could also be used as a proxy for measuring breastfeeding cessation, since we would expect milk volume to decrease (as a dependent variable) as a result of changing mother–infant breastfeeding dynamics. Data collection tools used to record milk volume over time might include self-report methodologies (such as maternal milk logs or pumping logs), digital records, or mobile phone applications (Johnson et al., 2022; Meier et al., 2013). Digital tools, in addition to data collection features, may have the capability to analyze milk volume data, summarize, or visualize potential reduction in maternal milk yield.

### Milk Transfer

Clinical researchers who conduct lactation assessments might measure breastfeeding cessation through measuring milk transfer, either through infant test weighing (Arthur et al., 1989) or through “dose-to-mother” deuterium dilution (Coward et al., 1982). Milk transfer measured through “test weighing” involves using a calibrated scale to measure the infant’s weight before and after direct breastfeeding. Maternal milk logs can also be used to monitor the transfer of milk by the infant over time.

The deuterium oxide “dose-to-mother” technique consists of providing the mother with a drink of deuterium-labelled water, which is excreted into human milk (Davidsson & Tanumihardjo, 2011). The disappearance of the deuterium from the mother and its enrichment in the infant can then be measured, for example, using saliva samples. Mathematical models are then used to calculate the total human milk transferred (Davidsson & Tanumihardjo, 2011; Scanlon et al., 2002).

In clinical practice, growth charts serve as a key tool during early infancy for monitoring infant growth and identifying growth faltering (when human milk intake and/or supplementation is insufficient to sustain normal infant growth). These tools enable practitioners and researchers to compare actual growth to growth percentiles in order to monitor if infant weight gain (or weight loss) is appropriate over time. Growth faltering may occur, for example, as a result of clinical breastfeeding difficulties. In these cases, infant growth may be used as a proxy for measuring premature breastfeeding cessation. Importantly, growth faltering may also occur in tandem with timely breastfeeding cessation if the child’s complementary foods or family diet are nutritionally inadequate. Hence, depending on the local context, infant and young child growth can be used as a proxy for measuring both premature and timely breastfeeding cessation.

### Feeding Schedules

In clinical practice, changes in feeding schedules may be used by parents or clinicians to gauge breastfeeding intensity. These may include changes in the number of nighttime or daytime feedings, or time intervals between feedings over a specified time frame. To our knowledge, there are no standardized measurement tools to assess a reduction in the intensity of breastfeeding based on infant feeding schedules alone. Table 1 provides examples of variables, methods, and tools for measuring breastfeeding cessation over time using maternal milk yield, milk transfer, and infant growth.

**Table 1.** Example Variables, Methods and Tools for Measuring Breastfeeding Cessation in Clinical Research.

Maternal Milk Yield	
Reduction in the volume of expressed human milk over a specified duration	Method: Monitoring human milk expression over time Tools: milk expression protocols to assess maternal milk yield over a period of time, including pumping or maternal milk logs, computer software, or mobile phone apps (Johnson et al., 2022; Kent et al., 2018; Lai et al., 2010; Meier et al., 2013; Roznowski et al., 2020) Instruments: breast pumps, bottle or syringe volume markers, calibrated food scales
Infant Milk Transfer	
Reduction in the volume of infant milk transferred over a specified duration	Method: Dose-to-the mother, deuterium oxide dilution (Coward et al., 1982) ○ Tool: IAEA Calculation Template for Breast Milk Intake (IAEA, 2010)
	Method: Infant test weighing (Arthur et al., 1987) ○ Tools: Calibrated infant scale to weigh infant before and after a breastfeeding occasion, maternal milk logs (Johnson et al., 2022; Meier et al., 2013), infant weighing sheets (Savenije, 2006)
Excessive infant weight loss over a specified duration	Method: Longitudinal infant weight monitoring Tools: Weight loss nomograms (Flaherman et al., 2013)
Infant Growth	
Infant growth faltering	Method: Plotting of anthropometric measurements over time Tools: Calibrated infant scale, heightometer, infantometer, circumference tapes, World Health Organization (WHO) growth velocity standards (WHO, 2009), Fenton growth charts (Fenton, 2013), Olsen growth curves (Olsen et al., 2010)

## Measurement of Breastfeeding Cessation in Public Health and Epidemiology

The field of public health emphasizes the protection and improvement of the health of populations, focusing on disease prevention and the promotion of well-being. Public health studies may generate data and insights that connect individual breastfeeding behaviors to broader systemic patterns, inform program and policy design, and highlight the sociocultural and structural determinants of breastfeeding behaviors across populations. From a policy and health systems perspective, evidence-based public health evaluates the feasibility and effectiveness of interventions, programs, and policies designed to support continued breastfeeding and prevent premature breastfeeding cessation. Epidemiology studies the distribution, patterns, and determinants of health and disease in populations. There are several ways of measuring breastfeeding cessation in populations over time.

In public health research, choosing which level of measurement to use for breastfeeding cessation is driven by the research question. Researchers may define breastfeeding cessation as a binary, single event at a definite time point (e.g., complete termination of human milk feeding at a specified time). This approach is useful in epidemiological disciplines applying time-to-event analyses (Theurich et al., 2025). Using this approach, breastfeeding attrition is calculated by dividing the number of breastfeeding dyads who stopped breastfeeding over a time period by the average number of breastfeeding dyads observed during that time period (Theurich et al., 2025).

In epidemiological surveillance, on the other hand, breastfeeding cessation may be measured and monitored to determine program effectiveness. For example, practitioners and researchers may monitor the ratio of the number of mothers still breastfeeding to all mothers in the study over a defined time period. An example is the WHO Indicators for Assessing Infant and Young Child Feeding Practices (e.g., percentage of children 12–23 months of age who were fed breast milk during the previous day; WHO & United Nations Children’s Fund [UNICEF], 2021). Large-scale cross-sectional and longitudinal surveys, such as the Infant Feeding Practices Study II (IFPS II), Pregnancy Risk Assessment Monitoring System (PRAMS), the Demographic and Health Surveys (DHS), and other national surveillance systems, serve as sources of data for tracking breastfeeding cessation over time (Haile, 2025). These datasets often include standardized breastfeeding indicators developed by organizations such as the U.S. Centers for Disease Control and Prevention (CDC), the WHO, and the UNICEF. Researchers can use these well-established indicators to assess breastfeeding prevalence, identify disparities by geography or socioeconomic status, and monitor trends across time and place. Geospatial tools and geographic information system (GIS) mapping have been utilized to identify spatio-temporal variations in premature breastfeeding cessation and identify breastfeeding service gaps (Aweke et al., 2025; Grubesic & Durbin, 2017; Tebeje et al., 2024; Yismaw et al., 2024). Finally, based on the data collected, statistical tools may be used to compare breastfeeding cessation in cohorts over time.

## Measurement Tools From Psychology

The field of psychology studies the human mind and human behavior. Through this lens, transitions in the states of breastfeeding and lactation are understood as biopsychosocial processes influenced by emotions, stress, bonding, cultural norms, and cognitive beliefs. A number of measurement tools from the field of psychology have been developed to measure parameters associated with breastfeeding cessation. Some of these tools were outlined in a previous editorial (Mazhar et al., 2025).

An example of child-initiated cessation of breastfeeding includes a “nursing strike,” defined as infant refusal to directly breastfeed or refusal of human milk without any apparent cause (Jalali et al., 2021). Examples of potential maternal psychological parameters related to breastfeeding cessation include: prenatal and postpartum intentions to breastfeed, maternal attitudes, knowledge and competencies, self-efficacy, personality, mental health, social support, satisfaction, self-esteem, and breastfeeding experiences (Asimaki et al., 2022; Wu et al., 2025). Other tools examine the role of bonding, caregivers, family dynamics, and social support systems, or the influence of intergenerational breastfeeding (Billings et al., 2024; Di Manno et al., 2015) in shaping transitions between breastfeeding states, including their influence on the cessation of breastfeeding. These tools acknowledge the breastfeeding dyad as a central unit of interaction, psychological attachment, and co-regulation. Psychometric measurement may be complemented by qualitative research (e.g., interviewing methods or focus group discussions), which serves to explore the lived experiences, perceptions, and challenges faced by breastfeeding mothers. Table 2 lists example measurement tools for maternal psychological parameters related to breastfeeding cessation.

**Table 2.** Example Tools for the Measurement of Maternal Psychological Parameters Relevant for Early Breastfeeding Cessation.

Example of Psychological Tools (in order of publication year)
• The Breastfeeding Attrition Prediction Tool (BAPT) (Janke, 1994) • Maternal Breastfeeding Evaluation Scale (MBES) (Leff et al., 1994) • Iowa Infant Feeding Attitude Scale (Mora et al., 1999) • Breastfeeding Self-Efficacy Scale (BSES) (Dennis, 1999) • Breastfeeding Self-Efficacy Scale – Short Form (BSES-SF) (Dennis, 2003) • Prenatal Breastfeeding Self-Efficacy Scale (P-BSES) (Wells et al., 2006) • Infant Feeding Intentions Scale (Nommsen-Rivers et al., 2010) • Breast Milk Expression Experience (BMEE) (Flaherman et al., 2013) • Breastfeeding Competency Scale (BCS) for pregnant women (Wu et al., 2021) • Breastfeeding Social Norms Scale (Charlesworth et al., 2023)

## Measurement Tools From Anthropology

Anthropology is the study of humanity, and within this discipline, there has been sustained interest in infant feeding practices. Anthropological understanding of human lactation focuses on both evolutionary and contemporary perspectives, and comes primarily from two subdisciplines biological anthropology and sociocultural anthropology—which encompass research from subfields such as biocultural anthropology, human biology, bioarcheology, medical anthropology, and primatology (Quinn et al., 2023; Sellen, 2007; Van Esterik, 2002). There has also been a concerted effort to uplift biocultural approaches to anthropological research on breastfeeding as it is both a physiological process and a socially-mediated, learned behavior (Quinn et al., 2023; Quinn, 2018; Van Esterik, 2002).

Biological anthropologists often use an evolutionary lens to understand how human biology, culture, and behaviors like breastfeeding evolved as humans adapted to environments around the world across time (Fewtrell et al., 2020). Theories such as life history theory, parental investment theory (Sellen, 2007), Trivers-Willard hypothesis (Wander & Mattison, 2013), and parent–infant conflict theory (Fouts et al., 2005) have been used by anthropologists to examine the evolutionary narrative of infant feeding, often centering the physiological balance of meeting both parental and offspring needs (Fewtrell et al., 2020; Quinn, 2018; Sellen, 2007). Biological anthropologists may use physiological measures to reconstruct breastfeeding patterns.

Methodologies such as isotope analysis of carbon, oxygen, and nitrogen within osteological, dental, and fossil remains provide evidence to support the age ranges for breastfeeding cessation in prehistoric populations (Dąbrowski et al., 2020; Stantis et al., 2020; Tsutaya, 2017; see Table 3). In contemporary populations, anthropologists are often interested in human variation, recognizing that a variety of social factors can shape our health and well-being. The collection of biomarker data is common among biological anthropologists and, in the context of lactation, has been used to signal physiological communication between parent and offspring (Hinde et al., 2015; Quinn et al., 2023). Finally, anthropometric measurements are also used, often to understand the relationship between breastfeeding and child growth and development (Miller, 2014; Moffat, 2001). Longitudinal measurement of these parameters may be helpful for pinpointing which factors influence parents or children to terminate breastfeeding, or which physiological signals or deficits may influence the timing of breastfeeding cessation.

**Table 3.** Examples of Anthropological Methods and Tools for Measuring Breastfeeding Cessation.

Population Type	Example Methods for Measuring Breastfeeding Cessation
Historic and prehistoric humans	Estimation of age at linear enamel hypoplasia formation using the Reid and Dean method (Reid & Dean, 2000) Isotope analysis from osteological remains (Stantis et al., 2020; Tsutaya, 2017) Isotope analysis from dental remains (Dąbrowski et al., 2020) Bayesian computational modeling (Stantis et al., 2020) Non-human primate models (Hinde et al., 2015) Biomarker collection (Hinde et al., 2015; Quinn et al., 2023) Anthropometry (Miller, 2014; Moffat, 2001)
Living populations	Ethnography and participant-observation (Fouts et al., 2005; Fouts et al., 2001, 2012) Interviews (e.g., semi-structured, go-along, life history, key informant) Focus group discussions Biomarker data Anthropometry

Ethnography and participant observation are foundational to research conducted by sociocultural anthropologists and are also employed by biocultural and medical anthropologists (Fouts et al., 2005; Fouts et al., 2001, 2012). Ethnography is the systematic study of a culture or group with the aim of understanding their social practices from the perspective of the members of the culture or group. Participant observation is a key method in ethnographic fieldwork, which involves getting close enough with a group of people to be allowed to observe and record their lives, providing the anthropologist with experiential knowledge on the topic(s) being studied (Bernard, 2017). These methods require the researcher to practice reflexivity, recognizing how their involvement shapes both their observations of participants’ practices, as well as their analysis of the culture or group (Salzman, 2002). Within the context of infant feeding, these methods have contributed to our understanding of breastfeeding cessation as a process, most often persisting over a period of time (Quinn, 2018), and heavily dictated by cultural norms and social processes (Dettwyler, 2004; Tsutaya & Mizushima, 2023). Additional qualitative methods used by anthropologists studying infant feeding practices include a variety of interviewing methods (e.g., semi-structured, go-along, life history, key informant), as well as focus group discussions. Surveying is also common, adding the ability for quantitative data collection of infant feeding practices (Fouts et al., 2012; Miller, 2014; Wander & Mattison, 2013) and allowing for mixed methods analyses to provide a more detailed understanding of the variation that exists across infant and young child feeding practices. Table 3 gives an overview of methods and tools for measuring breastfeeding cessation from the field of Anthropology.

While evolutionary forces have shaped a sensitive period where breastfeeding cessation strikes the balance between the growth and development of young children and maternal energy needs, anthropological research also demonstrates how cultural norms and social practices ultimately dictate the wide variation we see in breastfeeding cessation behaviors across human populations (Quinn et al., 2023; Quinn, 2018; Sellen, 2007).

## Qualitative and Quantitative Approaches for the Measurement of Breastfeeding Cessation

Across disciplines, breastfeeding cessation can be measured using qualitative or quantitative research approaches or may use mixed methodology. Researchers may draw from a variety of tools and instruments to collect data. Examples of qualitative methods for studying breastfeeding cessation are similar to other health research and might include interviewing, focus group discussions, or cultural domain analysis (Haile & Woldu, 2024), or photovoice (Burton et al., 2024). Examples of quantitative measurement of breastfeeding cessation might include surveys, maternal milk logs, mobile apps, or digital technologies for systematically collecting data on human milk provision over time. Table 4 presents examples of potential qualitative or quantitative research approaches that can be used to measure breastfeeding cessation.

**Table 4.** Potential Research Approaches for Measurement of Breastfeeding Cessation.

Type	Examples of Approaches for Measuring Breastfeeding Cessation
Qualitative	Research of maternal experiences during desired or undesired breastfeeding cessation Recording maternal breastfeeding goals using a verbal questionnaire (Hoban et al., 2015) Examining breastfeeding experiences in women with altered hormonal levels or gene mutations that affect their ability to reach their breastfeeding goals Discussions with parents or healthcare providers who support breastfeeding cessation after infant loss Patient-reported outcomes or side effects of drugs causing unintended lactation suppression Patient-reported experiences of breastfeeding cessation during contraindicated therapies
Quantitative	Research of maternal self-reported expressed human milk volumes in relationship to maternal predictors of premature breastfeeding cessation Quantification of expressed milk volumes and number of breastfeedings, according to maternal milk logs, in relationship to infant growth over time Measurement of mixed feeding or changes in the ratio of milk and breastmilk substitute volumes over time in dyads who wish to end the breastfeeding relationship (measurement of time until complete cessation) Quantification of volume of human milk intakes during breastfeeding cessation using doubly labeled water (Scanlon et al., 2002)

## Conclusion

This article contributes several methodological concepts on breastfeeding cessation. We highlight opportunities for more precise definitions of breastfeeding cessation in empirical literature and offer various tools for measuring it. Considerations for choosing the level of measurement, as it relates to the potential research question and scientific discipline, are given. Since the measurement tools outlined are not exhaustive, a more systematic approach to the search and evaluation of available measurement tools for breastfeeding cessation is likely warranted. Through improving awareness of the various concepts and measurement approaches, this article aims to improve methodological rigor and comparability across research studies investigating breastfeeding cessation.
